# Body size and allometric shape variation in the molly *Poecilia vivipara* along a gradient of salinity and predation

**DOI:** 10.1186/s12862-014-0251-7

**Published:** 2014-12-04

**Authors:** Márcio S Araújo, S Ivan Perez, Maria Julia C Magazoni, Ana C Petry

**Affiliations:** Departamento de Ecologia, Universidade Estadual Paulista, Rio Claro, SP Brazil; División Antropología, Facultad de Ciencias Naturales y Museo, Universidad Nacional de La Plata, CONICET, La Plata, Argentina; Núcleo em Ecologia e Desenvolvimento Socioambiental de Macaé – NUPEM, Universidade Federal do Rio de Janeiro – UFRJ, Macaé, RJ Brazil

**Keywords:** Divergent natural selection, Ecological gradients, *Hoplias malabaricus*, Morphometrics, Poeciliidae

## Abstract

**Background:**

Phenotypic diversity among populations may result from divergent natural selection acting directly on traits or via correlated responses to changes in other traits. One of the most frequent patterns of correlated response is the proportional change in the dimensions of anatomical traits associated with changes in growth or absolute size, known as allometry. Livebearing fishes subject to predation gradients have been shown to repeatedly evolve larger caudal peduncles and smaller cranial regions under high predation regimes. *Poecilia vivipara* is a livebearing fish commonly found in coastal lagoons in the north of the state of Rio de Janeiro, Brazil. Similar to what is observed in other predation gradients, lagoons inhabited by *P. vivipara* vary in the presence of piscivorous fishes; contrary to other poeciliid systems, populations of *P. vivipara* vary greatly in body size, which opens the possibility of strong allometric effects on shape variation. Here we investigated body shape diversification among six populations of *P. vivipara* along a predation gradient and its relationship with allometric trajectories within and among populations.

**Results:**

We found substantial body size variation and correlated shape changes among populations. Multivariate regression analysis showed that size variation among populations accounted for 66% of shape variation in females and 38% in males, suggesting that size is the most important dimension underlying shape variation among populations of *P. vivipara* in this system. Changes in the relative sizes of the caudal peduncle and cranial regions were only partly in line with predictions from divergent natural selection associated with predation regime.

**Conclusions:**

Our results suggest the possibility that adaptive shape variation among populations has been partly constrained by allometry in *P. vivipara*. Processes governing body size changes are therefore important in the diversification of this species. We conclude that in species characterized by substantial among-population differences in body size, ignoring allometric effects when investigating divergent natural selection’s role in phenotypic diversification might not be warranted.

**Electronic supplementary material:**

The online version of this article (doi:10.1186/s12862-014-0251-7) contains supplementary material, which is available to authorized users.

## Background

A central goal of research in evolutionary biology is to understand the factors involved in the origin and maintenance of phenotypic variation [1]. Divergent natural selection (DNS) – selection pulling trait means of populations towards different adaptive peaks – is usually believed to be a primary mechanism generating and maintaining adaptive phenotypic diversity among populations [2-4]. When populations are subject to different ecological pressures and there is sufficient standing genetic variation, DNS can act directly on phenotypic traits, sometimes leading to rapid phenotypic change [5,6].

Alternatively, phenotypic variation among populations may arise indirectly through correlated responses to selection on other traits [7,8]. In the case of morphology, correlated responses of traits can be a consequence of developmental interactions among structures and/or pleiotropic effects during development [9,10]. These interactions and/or pleiotropic effects are important because they might channel the direction and influence the pace of adaptive changes in complex morphological traits [11,12]. One of the most frequent patterns of correlated responses is the proportional change in the dimensions of particular anatomical traits associated with change in growth or absolute size, known as allometry [13]. Several studies have indicated that within-population allometric patterns can constrain changes in morphology among populations and species, so that size variation determines the direction of evolutionary change in morphological traits [14-17].

Livebearing fishes of the family Poeciliidae are important model organisms in the study of the effects of DNS on phenotypic variation [18]. For example, the strongest evidence available for the role of predation in driving phenotypic evolution in fishes comes from poeciliid fishes [19,20]. In particular, there is empirical evidence of trade-offs in swimming performance that lead to the divergence of body shape – which is highly heritable [21]– among populations subject to different levels of predation [21-25]. In the presence of predators, selection favors fast-start performance leading to the evolution of relatively larger caudal peduncles and smaller cranial regions, whereas in the absence of predators smaller caudal peduncles and larger cranial regions are favored as they reduce drag during steady swimming.

*Poecilia vivipara* is a small (2–5 cm body length) livebearing fish distributed along the Atlantic coast of South America (from Venezuela to La Plata River in Argentina) [26] and commonly found in coastal lagoons in the north of the state of Rio de Janeiro, Brazil [27]. Lagoons in this system vary in salinity [28] and in the presence of piscivorous fishes (e.g., the trahira *Hoplias malabaricus*), which are usually absent from lagoons with brackish to salt water [27,29]. One prior study in the area revealed that populations of *P. vivipara* inhabiting low predation environments had relatively smaller caudal regions than those in high predation environments [30], in line with previous findings on other poeciliids. One particular feature of this system is that *P. vivipara* shows remarkable variation in body size among populations, which is positively correlated with salinity [30-32]. Correlated responses of body shape to variation in body size have never been explored in this species though.

Here we investigate body shape diversification among populations of *P. vivipara* which differ substantially in body size and inhabit lagoons varying in the presence of piscivorous fishes and salinity [27]. Because populations differ in average body size, we investigate if shape variation among populations is chanelled by allometric effects [33]. We evaluated the effect of body size on body shape variation within and among populations (within-population and evolutionary allometry, respectively) by testing the relationship between shape and size variation using a multivariate regression model [13]. We then compared the within-population and evolutionary allometries to evaluate whether allometric shape changes among populations are aligned with the patterns of allometric shape change within populations. Assuming that predation pressure on *P. vivipara* is higher in lagoons containing piscivores, we expect populations to differ in body shape in line with predictions from trade-offs in swimming performance.

## Methods

### Field sampling

**Figure 1:**
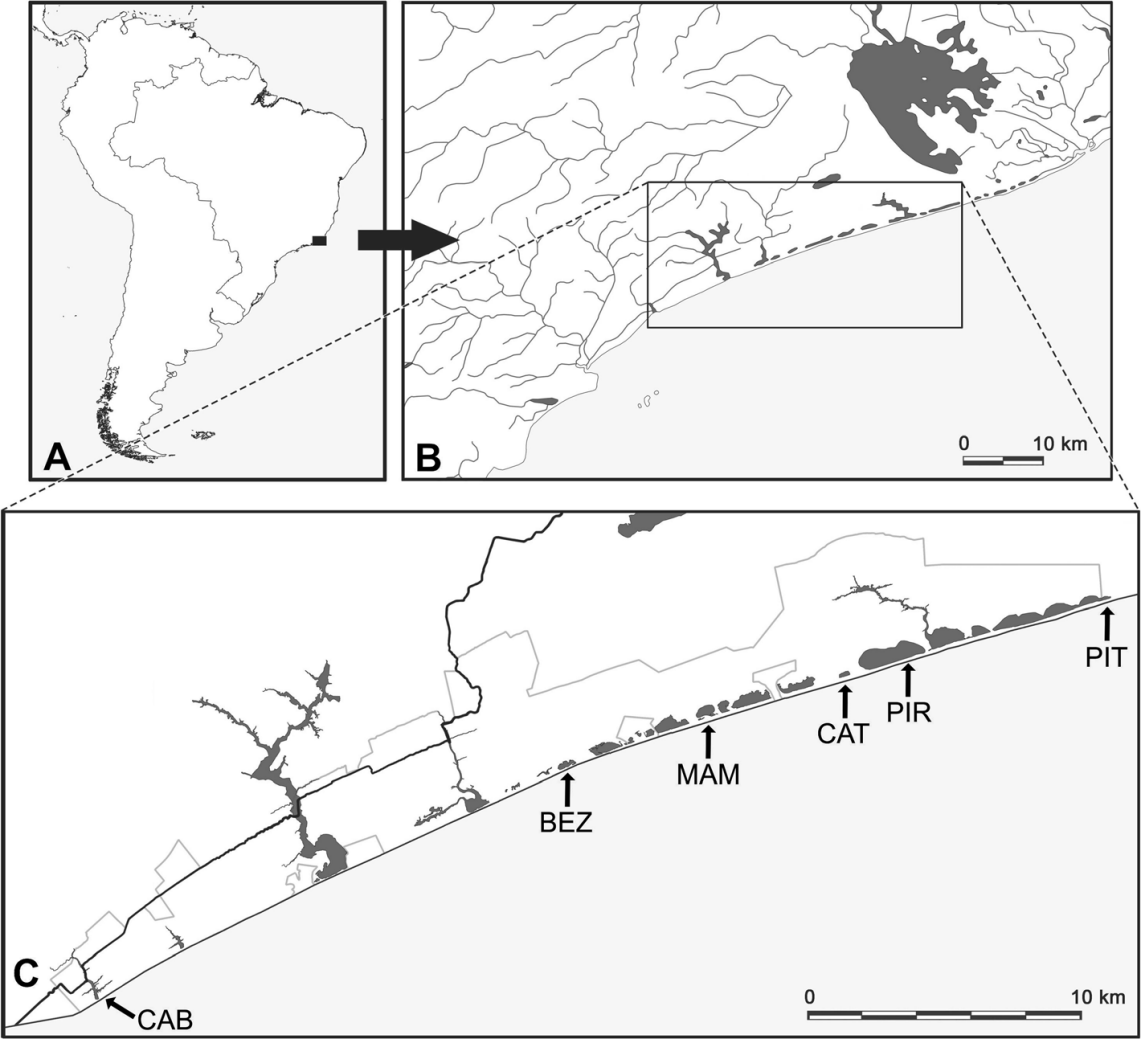
**Study area in northern state of Rio de Janeiro, Brazil (A) and location of the studied coastal lagoons (B-C).** Lagoon names are Cabiúnas (CAB), Bezerra (BEZ), Maria Menina (MAM), Catingosa (CAT), Pires (PIR), and Pitanga (PIT).

We analyzed specimens from six coastal lagoons in the Parque Nacional da Restinga de Jurubatiba (PARNA Jurubatiba), a relatively well-preserved portion of the coast of northern Rio de Janeiro State, Brazil, with 14,860 ha (Figure 1). Based on literature information [27] and preliminary field observations, we chose populations differing in body size and inhabiting lagoons with contrasting predation regimes. Specifically, we chose three lagoons harboring at least one species of piscivorous fish and three lagoons with no prior record of piscivorous species. Because ubiquitous piscivores in these lagoons are freshwater species, lagoons containing piscivores are at the lower end of the salinity gradient (Table 1). We selected lagoons that are temporally stable (i.e., for which there was no previous record of drying up in years of low precipitation) and isolated from each other and therefore treated our studied populations as independent. We acknowledge that in years of exceptionally high precipitation during the rainy season (more than 800 mm accumulated between October and March [34]) the neighbor lagoons Catingosa and Pires might eventually connect.

**Table 1:** **Average salinity of lagoons, number of piscivores, and sample sizes of** ***P. vivipara***

**Lagoon**	**Salinity (ppt)**	**Piscivores**			***P. vivipara***	
		***H. mala***	***H. uni***	***T. stri***	**Females**	**Males**
Cabiúnas (CAB)	0.15	13		23	76	46
Bezerra (BEZ)	2.17	16	2		123	53
Pitanga (PIT)	6.93				109	86
Catingosa (CAT)	22.03				98	45
Pires (PIR)	21.53				68	28
Maria Menina (MAM)	26.17				84	72

ppt: parts per thousand. *H. mala*: *Hoplias malabaricus*; *H. uni*: *Hoplerythrinus unitaeniatus*; *T. stri*: *Trachelyopterus striatulus*. Additional piscivore specimens were sampled with seine net in Cabiúnas (*H. mala* = 3; *T. stri* = 4; *Oligosarcus hepsetus* = 10); Bezerra (*H. mala* = 9); and Pitanga (*H. mala* = 1; *H. uni*: 15). Empty cells indicate zero.

Lagoons were surveyed four times from July 2011 to January 2013. In every survey, we measured salinity with a YSI-85 meter and quantified the abundance of piscivores with longlines holding 50 hooks baited with *P. vivipara* and two other species of livebearing fishes commonly found in the area (*Jenynsia multidentata* and *Phalloptychus januarius*). We placed one longline per lagoon at dusk and collected it the next morning (~15 hours; total sampling effort: ~3,000 hook-hours per lagoon), when captured piscivores were quantified (Table 1). Specimens of *P. vivipara* were collected with a seine net (1.5 m high, 5 m wide, mesh size: 0.5 mm), pulled by two of us near the shore (depth < 2 m). Upon collection, specimens were euthanized in Eugenol and fixed in 10% formalin. Specimens were later transfered to 70% ethanol in the laboratory. During sampling with the seine net we incidentally collected piscivores (sampling effort not standardized across lagoons), which were euthanized and preserved for further analysis. Gut-content analysis of these specimens indicates that these species are effective predators of *P. vivipara* in this system (A. C. Petry, unpub. data). This study was conducted under the Brazilian System of Authorization and Information on Biodiversity-SISBIO (permit #28136-2) and authorized by the Ethics Committee on Animal Use of the Universidade Estadual Paulista-CEUA-IB-UNESP-CRC (process #0502).

### Morphometric and Statistical Analyses

**Figure 2:**
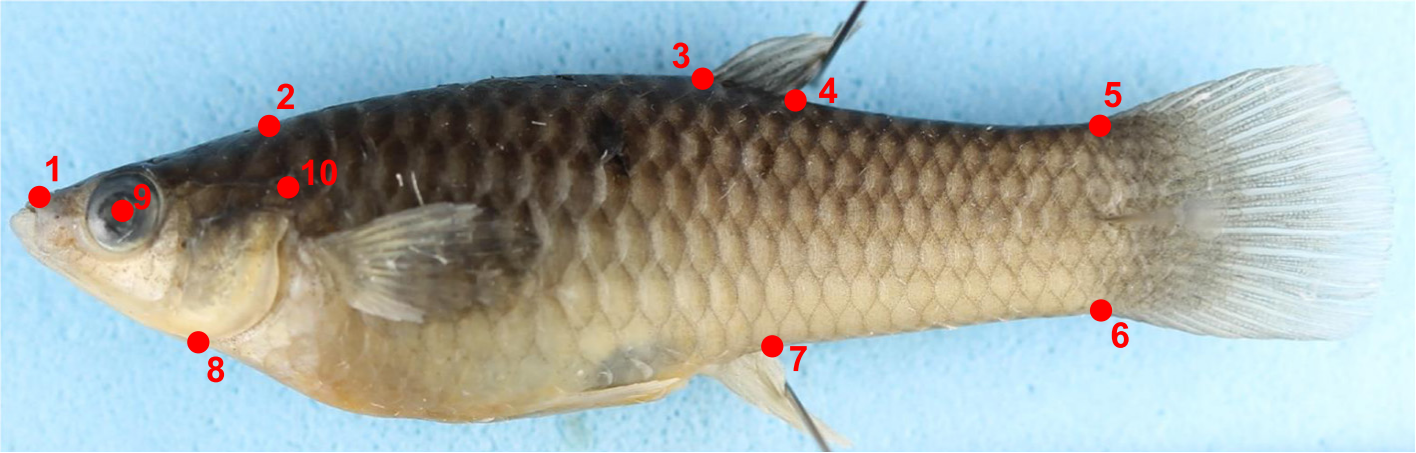
**Female** ***Poecilia vivipara*** **showing the body landmarks used in this study: (1) most anterodorsal point of premaxilla, (2) most posterodorsal point of skull, (3) anterior insertion of dorsal fin, (4) posterior insertion of dorsal fin, (5) dorsal insertion of caudal fin, (6) ventral insertion of caudal fin, (7) posterior insertion of anal fin, (8) most posteroventral point of skull, (9) center of the eye, and (10) most posterodorsal point of opercle [** 23 **,** 30 **].** Landmarks 4–7 delimit the caudal peduncle.

Body shape variation was analyzed with landmark-based morphometric techniques and multivariate statistical methods [35]. Morphology was captured from digital images as 2D Cartesian coordinates for landmarks in lateral view (Figure 2). Images were obtained with a DSLR Canon Rebel T3 camera with a macro 60 mm lens. Specimens were positioned in the lateral plane and the camera lens was placed parallel to the sagittal plane of each fish. The images were obtained at a 100 mm distance from the lateral line. After image capture, specimens were dissected and age class (juveniles vs. adults) and sex were determined by gonad inspection. Only adult individuals were analyzed (330 males and 558 females; Table 1).

The coordinates of 10 landmarks (Figure 2) were registered for each specimen, using the software tpsDIG 2.10 [36]. The intra-observer error associated with the register of point coordinates was evaluated using a random sample of 42 specimens by digitizing the same set of landmarks from the same images in two events two weeks apart from each other. We tested for differences between the two series using a PROTEST analysis [37], and found that series were highly correlated (Procrustes pseudo-correlation = 0.97, *P* < 0.001), indicating a high level of consistency in landmark digitalization.

We used the natural logarithm of the centroid size (CS), defined as the square root of the summed squared distances from all landmarks to the configuration centroid [35,38], as a body size measure. To test for differences in body size among populations, an analysis of variance (ANOVA) was performed using ln(CS) as the dependent variable and population of origin as the independent variable.

We aligned landmark coordinates using a Generalized Procrustes Analysis (GPA) [38]. This analysis optimally translates, scales and rotates coordinates of landmarks using a least squares criterion [35,38]. The coordinates aligned by this procedure are called Procrustes shape coordinates and were used as variables in the following multivariate statistical analyses. The main axes of shape variation were described using Principal Components Analysis (PCA) and Canonical Variates Analysis (CVA) obtained from the Procrustes shape coordinates [39]. Both analyses provide a low dimensional representation of shape space among specimens and populations. The PCA is a rigid rotation of the Procrustes shape coordinates that maximizes the variation among individuals using a spectral decomposition of a covariance matrix [39,40]. The CVA is a non-rigid transformation of the Procrustes shape coordinates that maximizes the ratio of the among-population sum of squares to the pooled within-population sum of squares using a spectral decomposition [39,41]. The patterns of shape change along CV scores were visualized using outline diagrams generated in MorphoJ 1.06a [42].

To test the significance of shape differences among populations, a multivariate analysis of variance (MANOVA) was performed using the first principal components (PCs) of Procrustes shape coordinates – summarizing more than 90% of shape variation; females: PC1-PC9; males: PC1-PC8 – as dependent variables and population of origin as the independent variable. We used the first PCs to reduce the dimension of the dependent matrix because of the large number of Procrustes shape variables [43].

Shape changes in landmark data associated with body size (the allometric shape vector) were described using the multivariate regression vector obtained from multivariate regressions (multivariate ordinary least squares [OLS] models) of the Procrustes shape variables on ln(CS) [15,17,44]. First, to evaluate the effect of size on shape differences among populations (i.e., evolutionary allometry), we studied the association between consensus Procrustes shape variables (the multivariate means of the Procrustes shape coordinates) and mean ln(CS) of each population using an OLS model and estimating the evolutionary allometric shape vector. Second, we estimated the effect of size on shape variation within populations (i.e., within-population allometry) for the pooled within-population data (i.e., the matrix that jointly estimates the common covariation pattern within several groups using the covariance matrices based on the mean centered Procrustes coordinates for each population) using a multivariate OLS model and estimating the within-population allometric shape vector. Using a pooled within-population regression requires that within-population allometries are sufficiently similar, so that the pooled within-group vector makes biological sense. We tested for the agreement between within-population allometries by comparing the mean angle between regression vectors with a parametric distribution to test the null hypothesis that the vectors have random directions in regression space. The angles between regression vectors were calculated as the arc-cosine of the inner product of regression vectors standardized to unit lengths [45,46]. The magnitude of size-related shape changes (degree of allometry) was measured as the proportion of total variation – in units of squared Procrustes distance – explained by the regressions. Evolutionary and within-population allometries were visualized by using outline diagrams obtained from the regression of Procrustes shape coordinates on ln(CS). Finally, we tested the agreement between evolutionary and within-population allometries by estimating the angles between evolutionary and within-population regression vectors and comparing the observed angles with a parametric distribution to test the null hypothesis that the vectors have random directions in regression space. Procrustes superimposition, CVA, and PCA were done in MorphoJ 1.06a; the PROTEST analysis was done in the package vegan of R 3.0.3 [47]; and ANOVAs and MANOVAs were done in SYSTAT 11 (Systat Software, Inc., San Jose, CA).

## Results

**Figure 3:**
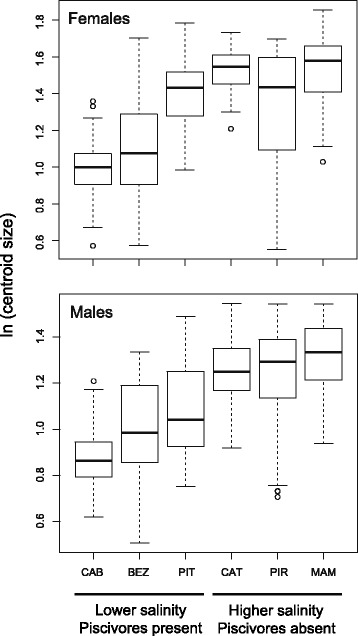
**Boxplot showing patterns of body size variation, measured using natural logarithm of the centroid size (CS), for females and males (median ± quartiles; whiskers: data range; dots: outliers).** Population names follow Figure 1.

We found substantial size variation both within and among populations (Figure 3). Overall, populations inhabiting environments with lower salinities and piscivores showed smaller average body size than populations living in higher salinities, where piscivores are absent. The ANOVA indicates that these differences are highly significant for females (*F* = 98.262, *P* < 0.001) and males (*F* = 41.485, *P* < 0.001).

**Figure 4:**
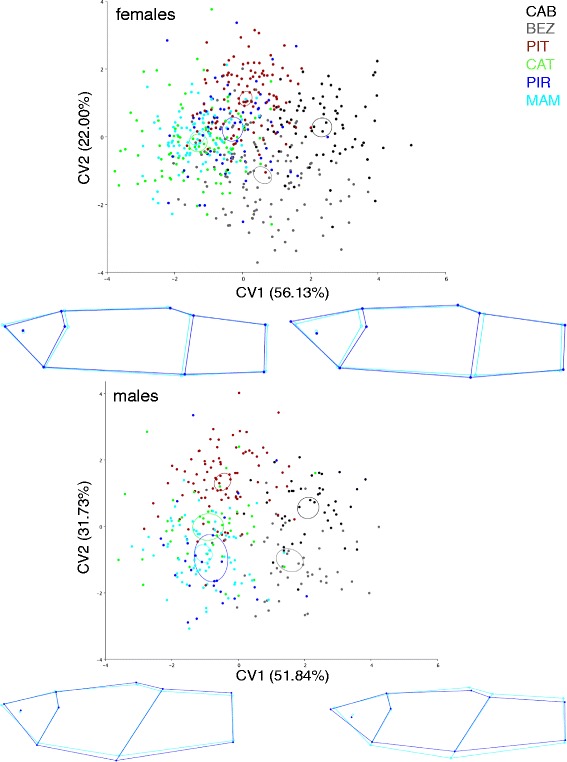
**CVA ordination showing the axis of major shape variation among the six populations studied.** Shape variation associated with CV1 is shown by wireframe changes (dark blue) in relation to the consensus configuration (light blue). Deformations presented correspond to the range of the CV1 axes. Ellipses represent 95% confidence intervals around population averages.

Body shape also varied among populations, as indicated by the first CV axis (ca. 50% of total variation), in which populations from lower salinity/high predation environments had higher average scores than those from higher salinity/low predation environments (Figure 4). For females, relatively longer caudal peduncles and larger cranial regions were associated with increasing CV1 scores (Figure 4). For males, larger CV1 scores were associated with a shallower caudal peduncle and a relatively larger and more pointy cranial region (Figure 4). The MANOVAs indicate that shape differences among populations are highly significant for both sexes (Females: Wilks’ Lambda = 0.386, *F* = 12.839, *P* < 0.001; Males: Wilks' Lambda = 0.334, *F* = 9.911, *P* < 0.001).

**Figure 5:**
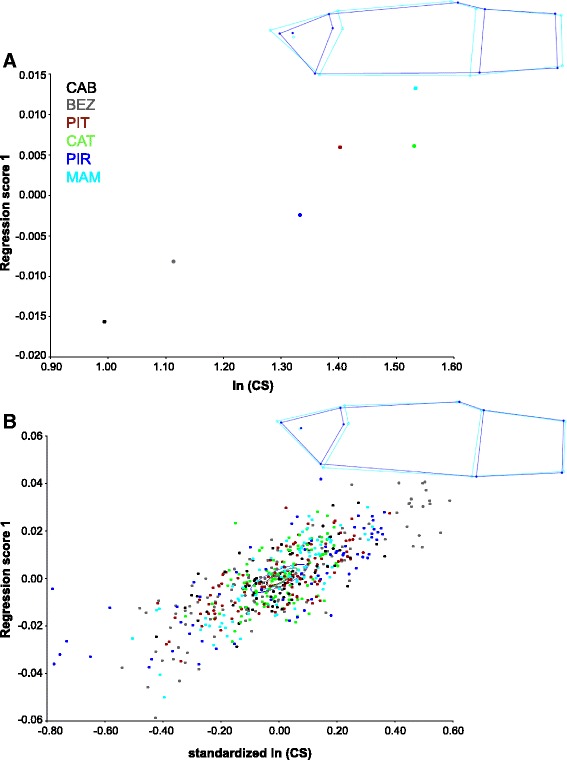
**Evolutionary and pooled within-population allometry in female body shape.** The wireframes show the changes in shape in relation to the consensus configuration (light blue) as ln (centroid size) increases. The deformations presented (dark blue) correspond to ln (centroid size) = 1.6 (evolutionary allometry) and ln (centroid size) = 0.6 (within-population allometry). The x-axes represent changes in centroid size (CS), whereas the y-axes display allometric shape (regression scores). Note that in panel **A** centroid size is in ln scale, whereas in panel **B** ln(CS) is standardized.

**Figure 6:**
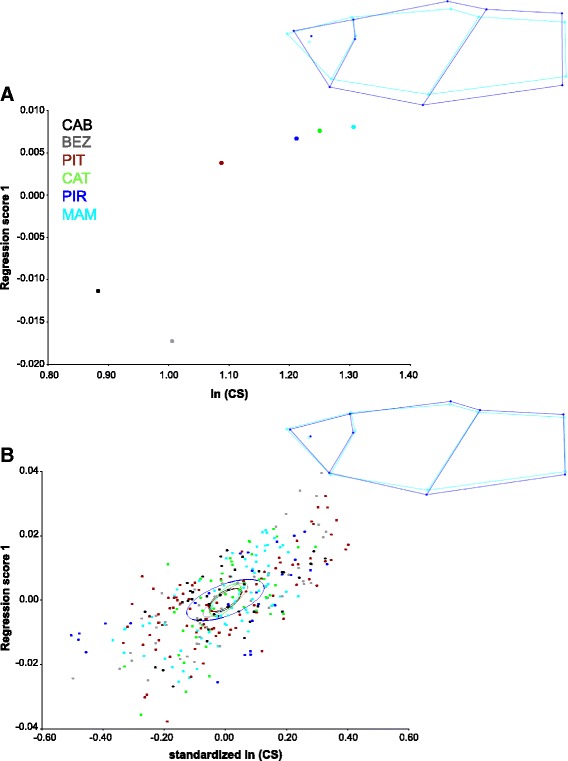
**Evolutionary and pooled within-population allometry in male body shape.** The wireframes show the changes in shape in relation to the consensus configuration (light blue) as ln (centroid size) increases. The deformations presented (dark blue) correspond to ln (centroid size) = 1.4 (evolutionary allometry) and ln (centroid size) = 0.6 (within-population allometry). The x-axes represent changes in centroid size (CS), whereas the y-axes display allometric shape (regression scores). Note that in panel **A** centroid size is in ln scale, whereas in panel **B** ln(CS) is standardized.

Body shape variation among populations was significantly related to size variation (Figures 5 and 6). The multivariate regression of Procrustes shape coordinates of population consensus on ln(CS) accounted for 65.69% of total shape variation for females (Figure 5A) and 38.35% for males (Figure 6A). The main shape changes along the regression vectors are similar to those observed in the CVA. For females, a shorter caudal peduncle and a smaller cranial region were observed in the populations with larger mean centroid size. For males, a relatively deeper body was observed in the populations with larger mean centroid size. The pooled within-population multivariate regression of Procrustes shape coordinates on ln(CS) indicated that 14.66% and 6.72% of the variation in body shape is associated with size for females and males, respectively. Shape changes associated with body size (allometric trajectories) within populations were similar among populations. We rejected the null hypothesis that within-population regression vectors have random directions in the regression space (Females: mean angle = 35 degrees, *P* < 0.001; Males: mean angle = 55 degrees, *P* < 0.001), indicating that within-population allometric vectors are sufficiently similar to compute one common allometric pattern across all populations.

The patterns of within-population shape changes were similar to those observed among populations (Figures 5 and 6). The angles between regression vectors of evolutionary and pooled within-population allometries were significantly smaller than would be expected if regression vectors had random directions in the regression space (Females: angle = 25 degrees, *P* < 0.001; Males: angle = 38 degrees, *P* < 0.001), indicating that within-population and evolutionary allometric trajectories have similar directions.

## Discussion

The populations studied showed substantial body size and correlated shape variation. Size variation among populations accounted for 66% of shape variation in females and 38% in males, suggesting that size is the most important dimension behind shape variation among *P. vivipara* in this system. Moreover, allometric shape changes among populations are aligned with allometric variation within populations, indicating that shape variation among populations is an indirect response to changes in body size. Our results, therefore, suggest that processes governing body size changes are important in the diversification of body shape in *P. vivipara*.

Populations of *P. vivipara* show a positive association between body size and salinity in this system [this study; 30,32]. The mechanisms underlying this trend are not yet clear and we can only speculate at this point. A previous experimental study on the congener *Poecilia latipinna* showed a high degree of plasticity in life-history traits, in which individuals grew faster and attained larger body sizes in saltwater than in freshwater [48]. In line with these findings, *P. latipinna* and *Gambusia affinis* showed larger body size and better body condition in brackish versus freshwater natural environments, suggesting that the latter is physiologically more stressful than the former for these species [49]. Additionally, a common garden experiment with two populations of *P. vivipara* inhabiting lagoons with contrasting salinities indicates that populations from brackish environments grow faster and reach larger body sizes than those from freshwater environments and that these differences are partly heritable [50], suggesting that populations might be genetically adapted to different salinity environments [51,52].

Another mechanism that could also account for variation in life-history traits among populations is predation regime. It is known that prey species can grow larger to avoid predation, because larger prey can be more difficult to be seized and/or consumed by their predators [53]. *Poecilia vivipara* is a relatively small fish (adult standard length = 1.3-5.6 cm in a sample of *N* = 2007 individuals); its main piscivore predator in this system is the trahira *H. malabaricus*, whose average adult standard length is 17,6 cm ± 7,14 (SD) in this system (A. C. Petry, unpub. data), and can easily prey on fish up to 50% its length [54,55]. It is therefore unlikely that *P. vivipara* can escape predation from *H. malabaricus* by growing larger body sizes. Alternatively, life-history theory predicts the evolution of larger body sizes at maturity in low-mortality environments [56], in line with our observation of larger body sizes in the high-salinity lagoons, where piscivores are absent.

Finally, differences in body size or growth rate might result from differences in energy intake determined by competition for food and/or resource productivity which happened to covary with salinity or predation regime. Preliminary data (M. S. Araújo, unpub. data) indicate no relationship between productivity and salinity in the studied lagoons. Additionally, the two potential competitors of *P. vivipara* in this system – the Cyprinodontiformes *J. multidentata* and *P. januarius* – are more abundant in the higher-salinity lagoons (unpublished results), precisely where *P. vivipara* attains larger body sizes, which suggests that interspecific competition is weak, if present at all.

Numerous examples among poeciliids suggest the evolution of optimal body shapes as an adaptive response to gradients of predation [22-25,30,57]. Specifically, there is a general trend of populations free from predation to present a relatively smaller caudal region and larger cranial region, because this body shape minimizes drag during steady swimming and, as a consequence, is favored by selection [21,58]. The opposite configuration (relatively larger caudal region and smaller cranial region), on the other hand, is favored where predators are present, because it maximizes fast starts and increases survivorship. Our results contradict to some extent this expectation: we found a trend of females living in piscivore-free environments to show relatively smaller caudal and cranial regions (where larger cranial regions would be expected) and males to show relatively larger, deeper caudal peduncles (where smaller caudal peduncles would be expected; Figure 4). A parsimonious explanation for this apparent conflict between this ecomorphological paradigm and our results is that predation by piscivores is not a selective agent in our study system. Several lines of evidence suggest that this is unlikely, though. First, prior work in other poeciliid fishes demonstrate increased mortality rates in the presence of piscivorous fishes [59-61]. Second, gut-content analysis of the piscivorous species in our system indicates that they are effective predators of *P. vivipara*; and the trahira *Hoplias malabaricus*, the most abundant piscivore in our sample (Table 1), has been shown to exert strong top-down control on its prey [62], including livebearing fish [63]. Finally, a previous study on the same system found evidence of natural selection for relatively larger caudal peduncles of *P. vivipara* in high-predation environments [30,31], suggesting that predation acts as an evolutionary force in this system.

Our results can be reconciled with previous findings if we recall that body shape has allometric and non-allometric components [35]. In prior work on poeciliids, including *P. vivipara*, researchers have mathematically removed linear allometric effects and focused on the non-allometric component of shape, whereas in our study we analyzed total shape (which includes both components). For the sake of comparison with previous studies, we analyzed the non-allometric component of shape using CVA. These results show a trend of larger caudal peduncles and smaller cranial regions in populations subject to predation (Additional file 1: Figures S1 and S2), in accordance with the ecomorphological paradigm proposed to explain the patterns of phenotypic variation in poeciliids [64]. It is worth noting that the non-allometric component of body shape only explained ca. 24% and 12% of total shape variation in females and males, respectively, compared to the 66 and 38% of shape variation associated with body size changes.

Assuming that these morphological syndromes – relatively larger head and smaller caudal peduncle versus relatively smaller head and larger caudal peduncle – represent adaptive peaks in this system, our results suggest the possibility that allometry prevented populations from attaining these optimal body shapes. The evolution of optimal body shapes in this case would require the evolution of allometric covariation structure [33], so that the direction of the selection gradient caused by predation and that of allometries would become aligned [65]. This should involve changes in the developmental-genetic basis of allometric trajectories so that the caudal region would become relatively smaller and the cranial region relatively larger during ontogeny (see Figures 5 and 6) – which could be achieved, for example, if growth of the cranial region outpaced growth of the caudal region during development. Theoretical and empirical evidence suggest that such changes in morphological integration – which necessarily involve changes in patterns of genetic covariance among traits – would require long time scales (e.g., millions of generations [7,11,65]; but see [66]). Geological evidence indicates that the most recent formation of this lagoon system occurred after the last marine ingression ca. 5,000 to 7,000 years ago [67]. Assuming that *P. vivipara* has 1–2 generations per year [6], these populations have been evolving for 2,500-7,000 generations. It is thus unlikely that there has been enough time for selection to change allometric trajectories so that populations could reach their adaptive peaks.

We acknowledge that our conclusions are based on the assumption that the ecomorphological paradigm proposed for poeciliids inhabiting predation gradients is an accurate description of the adaptive landscape in the studied system. This assumption is based on *a priori* predictions derived from theory on swimming functional morphology which were largely tested in numerous empirical examples showing the evolution of body shape in poeciliids inhabiting predation gradients. Alternatively, it is also plausible that other selective agents (e.g. salinity, feeding) also act on the studied populations and that the observed variation in body shape is actually a compromise between these multifarious selective agents. At the present moment it is not possible to determine if allometric trajectories indeed evolved and populations actually reached their adaptive peaks along this ecological gradient or if allometry has acted as a constraint preventing populations from reaching their adaptive peaks. Future investigations on the role of other putative selective agents on the evolution of body size and shape in this system might shed light on the issue.

## Conclusions

Poeciliid fishes have been used as a model system to demonstrate that populations subject to divergent natural selection may repeatedly evolve similar solutions to similar problems [64]. Although these shared responses to selection might be a common feature of phenotypic evolution in poeciliids and organisms in general, our results suggest the possibility that allometric effects, by chanelling shape change, might prevent populations from reaching their adaptive peaks. Therefore, in organisms characterized by substantial among-population differences in body size, it might not be safe to ignore allometric effects when investigating divergent natural selection’s role in phenotypic diversification.

## Availability of supporting data

The data set supporting the results of this article is available in the Dryad repository, [http://dx.doi.org/10.5061/dryad.4h31p]. [68].

## Additional file

Additional file 1: Figures S1 and S2. CVA ordinations showing the axes of major non-allometric shape variation.

## References

[1] Barton NH, Briggs DE, Eisen JA, Goldstein DB, Patel NH (2007). Evolution.

[2] Endler JA (1977). Geographic Variation, Speciation, and Clines.

[3] Schluter D (2000). The Ecology of Adaptive Radiation.

[4] Schluter D (2009). Evidence for Ecological Speciation and Its Alternative. Science.

[5] Carroll SP, Hendry AP, Reznick DN, Fox CW (2007). Evolution on ecological time-scales. Funct Ecol.

[6] Reznick DN, Shaw FH, Rodd FH, Shaw RG (1997). Evaluation of the rate of evolution in natural populations of guppies (*Poecilia reticulata*). Science.

[7] Lande R (1979). Quantitative genetic analysis of multivariate evolution, applied to brain: body size allometry. Evolution.

[8] Cheverud JM (1982). Phenotypic, genetic, and environmental morphological integration in the cranium. Evolution.

[9] Cheverud JM (1996). Developmental integration and the evolution of pleiotropy. Am Zool.

[10] Shingleton AW, Frankino WA, Flatt T, Nijhout HF, Emlen DJ (2007). Size and shape: the developmental regulation of static allometry in insects. BioEssays.

[11] Schluter D (1996). Adaptive radiation along genetic lines of least resistance. Evolution.

[12] Klingenberg C (2010). There’s something afoot in the evolution of ontogenies. BMC Evol Biol.

[13] Klingenberg C (1998). Heterochrony and allometry: the analysis of evolutionary change in ontogeny. Biol Rev.

[14] Marroig G, Cheverud JM (2001). A comparison of phenotypic variation and covariation patterns and the role of phylogeny, ecology, and ontogeny during cranial evolution of New World monkeys. Evolution.

[15] Drake AG, Klingenberg CP (2008). The pace of morphological change: historical transformation of skull shape in St Bernard dogs. Proc R Soc B Biol Sci.

[16] Mitteroecker P, Gunz P, Bernhard M, Schaefer K, Bookstein FL (2004). Comparison of cranial ontogenetic trajectories among great apes and humans. J Hum Evol.

[17] Gonzalez PN, Perez SI, Bernal V (2011). Ontogenetic allometry and cranial shape diversification among human populations from South America. Anat Rec Adv Integr Anat Evol Biol.

[18] Langerhans RB (2010). Predicting evolution with generalized models of divergent selection: a case study with poeciliid fish. Integr Comp Biol.

[19] Endler JA (1995). Multiple-trait coevolution and environmental gradients in guppies. Trends Ecol Evol.

[20] Reznick DN, Ghalambor CK, Crooks K (2008). Experimental studies of evolution in guppies: a model for understanding the evolutionary consequences of predator removal in natural communities. Mol Ecol.

[21] Langerhans RB (2009). Trade-off between steady and unsteady swimming underlies predator-driven divergence in Gambusia affinis. J Evol Biol.

[22] Langerhans RB (2009). Morphology, performance, fitness: functional insight into a post-Pleistocene radiation of mosquitofish. Biol Lett.

[23] Langerhans RB, Gifford ME, Joseph EO (2007). Ecological speciation in *Gambusia* fishes. Evolution.

[24] Langerhans RB, Layman CA, Shokrollahi AM, DeWitt TJ (2004). Predator-driven phenotypic diversification in *Gambusia affinis*. Evolution.

[25] Langerhans RB, Makowicz AM (2009). Shared and unique features of morphological differentiation between predator regimes in *Gambusia caymanensis*. J Evol Biol.

[26] Lucinda P, Reis RE, Kullander SO, Ferraris CJ (2003). Family Poeciliidae. Check List of the Freshwater Fishes of South and Central America.

[27] Di Dario F, Petry AC, Pereira MMS, Mincarone MM, Agostinho LS, Camara EM, Caramaschi EP, Britto MR (2013). An update on the fish composition (Teleostei) of the coastal lagoons of the Restinga de Jurubatiba national park and the Imboassica lagoon, northern Rio de Janeiro state. Acta Limnologica Brasiliensia.

[28] Caliman A, Carneiro LS, Santangelo JM, Guariento RD, Pires APF, Suhett AL, Quesado LB, Scofield V, Fonte ES, Lopes PM, Sanches LF, Azevedo FD, Marinho CC, Bozelli RL, Esteves FA, Farjalla VF (2010). Temporal coherence among tropical coastal lagoons: a search for patterns and mechanisms. Braz J Biol.

[29] Caramaschi EP, Sanchez-Botero JI, Hollanda-Carvalho P, Brandão CAS, Soares CL, Novaes JLC, Bartolette R, Rocha CFD, Esteves FA, Scarano FR (2004). Peixes das Lagoas Costeiras do Norte Fluminense: Estudos de Caso. Pesquisas de Longa Duração na Restinga de Jurubatiba: Ecologia, História Natural e Conservação.

[30] Gomes JL, Monteiro LR (2008). Morphological divergence patterns among populations of *Poecilia vivipara* (Teleostei Poeciliidae): test of an ecomorphological paradigm. Biol J Linn Soc Lond.

[31] Monteiro LR, Gomes JL (2005). Morphological divergence rate tests for natural selection: uncertainty of parameter estimation and robustness of results. Genet Mol Biol.

[32] Neves FM, Monteiro LR (2003). Body shape and size divergence among populations of *Poecilia vivipara* in coastal lagoons of south-eastern Brazil. J Fish Biol.

[33] Klingenberg C (2010). Evolution and development of shape: integrating quantitative approaches. Nat Rev Genet.

[34] INMET - Instituto Nacional de Meteorologia: *Estação Campos.* Brasil: Ministério da Agricultura, Pecuária e Abastecimento. http://www.inmet.gov.br.

[35] Mitteroecker P, Gunz P (2009). Advances in geometric morphometrics. Evol Biol.

[36] Rohlf FJ: **TpsDig2.** 2008. available at http://life.bio.sunysb.edu/morph.

[37] Peres-Neto P, Jackson D (2001). How well do multivariate data sets match? The advantages of a Procrustean superimposition approach over the Mantel test. Oecologia.

[38] Rohlf FJ, Slice DE (1990). Extensions of the procrustes method for the optimal superimposition of landmarks. Syst Zool.

[39] Mitteroecker P, Bookstein F (2011). Linear discrimination, ordination, and the visualization of selection gradients in modern morphometrics. Evol Biol.

[40] Rohlf FJ (1993). Relative warp analysis and an example of its application to mosquito wings. Contributions Morphometrics.

[41] Albrecht GH (1980). Multivariate analysis and the study of form, with special reference to canonical variate analysis. Am Zool.

[42] Klingenberg CP (2011). MorphoJ: an integrated software package for geometric morphometrics. Mol Ecol Resour.

[43] Sheets HD, Covino KM, Panasiewicz JM, Morris SR (2006). Comparison of geometric morphometric outline methods in the discrimination of age-related differences in feather shape. Front Zool.

[44] Mitteroecker P, Gunz P, Bookstein FL (2005). Heterochrony and geometric morphometrics: a comparison of cranial growth in *Pan paniscus* versus *Pan troglodytes*. Evol Dev.

[45] Blackith RE, Reyment RA (1971). Multivariate Morphometrics.

[46] Klingenberg CP, Marugán-Lobón J (2013). Evolutionary covariation in geometric morphometric data: analyzing integration, modularity, and allometry in a phylogenetic context. Syst Biol.

[47] R Development Core Team (2014). R: A Language and Environment for Statistical Computing.

[48] Trexler JC, Travis J (1990). Phenotypic plasticity in the sailfin molly, Poecilia latipinna (Pisces: Poeciliidae). I. Field experiments. Evolution.

[49] Martin SB, Hitch AT, Purcell KM, Klerks PL, Leberg PL (2009). Life history variation along a salinity gradient in coastal marshes. Aquat Biol.

[50] Araújo LG, Monteiro LR (2013). Growth pattern and survival in populations of *Poecilia vivipara* (Teleostei; Poeciliidae) inhabiting an environmental gradient: a common garden study. Environ Biol Fish.

[51] Shikano T, Fujio Y (1998). Strain differences in seawater adaptability in newborn guppy *Poecilia reticulata*. Fish Sci.

[52] Purcell KM, Hitch AT, Klerks PL, Leberg PL (2008). Adaptation as a potential response to sea-level rise: a genetic basis for salinity tolerance in populations of a coastal marsh fish. Evol Appl.

[53] Langerhans RB, Elewa AMT (2006). Evolutionary consequences of predation: avoidance, escape, reproduction, and diversification. Predation in Organisms: A Distinct Phenomenon.

[54] Vd A, Hahn N, Vazzoler AM (1997). Feeding patterns in five predatory fishes of the high Paraná River floodplain (PR, Brazil). Ecol Freshw Fish.

[55] de los Angeles Bistoni M, Haro JG, Gutiérrez M (1995). Feeding of *Hoplias malabaricus* in the wetlands of Dulce river (Córdoba, Argentina). Hydrobiologia.

[56] Stearns SC (1992). The Evolution of Life Histories, vol. 249.

[57] Tobler M, DeWitt TJ, Schlupp I, de León FJ G, Herrmann R, Feulner PG, Tiedemann R, Plath M (2008). Toxic hydrogen sulfide and dark caves: phenotypic and genetic divergence across two abiotic environmental gradients in *Poecilia mexicana*. Evolution.

[58] Langerhans RB, Reznick DN, Domenici P, Kapoor BG (2009). Ecology and evolution of swimming performance in fishes: predicting evolution with biomechanics. Fish Locomotion: An Etho-ecological Perspective.

[59] Heinen J, Coco M, Marcuard M, White D, Peterson MN, Martin R, Langerhans RB (2013). Environmental drivers of demographics, habitat use, and behavior during a post-Pleistocene radiation of Bahamas mosquitofish (*Gambusia hubbsi*). Evol Ecol.

[60] Reznick D, Bryant M (2007). Comparative long-term mark-recapture studies of guppies (*Poecilia reticulata*): differences among high and low predation localities in growth and survival. Ann Zool Fenn.

[61] Johnson JB, Zúñiga-Vega JJ (2009). Differential mortality drives life-history evolution and population dynamics in the fish *Brachyrhaphis rhabdophora*. Ecology.

[62] Petry AC, Gomes LC, Piana PA, Agostinho AA (2010). The role of the predatory trahira (Pisces: Erythrinidae) in structuring fish assemblages in lakes of a Neotropical floodplain. Hydrobiologia.

[63] Mazzeo N, Iglesias C, Teixeira-de Mello F, Borthagaray A, Fosalba C, Ballabio R, Larrea D, Vilches J, Garcia S, Pacheco JP, Jeppesen E (2010). Trophic cascade effects of *Hoplias malabaricus* (Characiformes, Erythrinidae) in subtropical lakes food webs: a mesocosm approach. Hydrobiologia.

[64] Langerhans RB, DeWitt TJ (2004). Shared and unique features of evolutionary diversification. Am Nat.

[65] Marroig G, Cheverud J (2005). Size as a line of least evolutionary resistance: diet and adaptive morphological radiation in New World monkeys. Evolution.

[66] Berner D, Stutz WE, Bolnick DI (2010). Foraging trait (co)variances in stickleback evolve deterministically and do not predict trajectories of adaptive diversification. Evolution.

[67] Martin L, Suguio K, Dominguez JML, Flexor JM (1997). Geologia do quaternário costeiro do litoral Norte do Rio de Janeiro e do Espírito Santo.

[68] Araújo MS, Perez SI, Magazoni MJC, Petry AC: **Data from: Body size and allometric shape variation in the molly*****Poecilia vivipara*****along a gradient of salinity and predation.** *Dryad Digital Repository*. http://dx.doi.org/10.5061/dryad.4h31p. 10.1186/s12862-014-0251-7 PMC4272540 25471469

