# Supplementary material for: Body size and allometric shape variation in the molly Poecilia vivipara along a gradient of salinity and predation
Source: BMC Evol Biol. 2014 Dec 4;14:251. doi: 10.1186/s12862-014-0251-7 (PMC4272540; doi:10.1186/s12862-014-0251-7)
Supplement: Additional file 1: Figures S1 and S2. — CVA ordinations showing the axes of major non-allometric shape variation. [file 12862_2014_251_MOESM1_ESM.doc]

**CVA ordinations showing the axes of major non-allometric shape variation**

We explored the main vectors of shape differences between populations using a Canonical Variates Analysis (CVA) obtained from the non-allometric shape variables . We defined the non-allometric shape space as the residuals of a multivariate OLS regression of Procrustes shape coordinates on ln (centroid size) for all populations . Non-allometric body shape varied among populations, as indicated by the first two CV axes (ca. 70% of non-allometric variation for females and males; Figs. S1 and S2). Females from high predation environments (BEZ, CAB, PIT) had higher average positive scores than those from low predation environments (CAT, MAM, PIR) on both CV axes, showing deeper caudal peduncles and smaller cranial regions (Fig. S1). For males, CV 2 showed a clear separation between predation regimes, in which larger scores were associated with deeper caudal peduncles and smaller cranial regions (Fig. S2). These trends are in line with predictions from trade-offs in swimming performance in response to predation gradients .

1. Mitteroecker P, Gunz P, Bernhard M, Schaefer K, Bookstein FL: **Comparison of cranial ontogenetic trajectories among great apes and humans**. *Journal of Human Evolution* 2004, **46**(6):679-698.

2. Drake AG, Klingenberg CP: **The pace of morphological change: historical transformation of skull shape in St Bernard dogs**. *Proceedings of the Royal Society B: Biological Sciences* 2008, **275**(1630):71-76.

3. Gonzalez PN, Perez SI, Bernal V: **Ontogenetic allometry and cranial shape diversification among human populations from South America**. *The Anatomical Record: Advances in Integrative Anatomy and Evolutionary Biology* 2011, **294**(11):1864-1874.

4. Langerhans RB, Reznick DN: **Ecology and evolution of swimming performance in fishes: predicting evolution with biomechanics**. In: *Fish locomotion: an etho-ecological perspective.* Edited by Domenici P, Kapoor BG. Enfield: Science Publishers; 2009: 200-248.

Figure S1. CVA ordination showing the axis of major non-allometric shape variation among females. Shape variation associated with CV1 and CV2 is shown by wireframe changes in relation to consensus configuration.

Figure S2. CVA ordination showing the axis of major non-allometric shape variation among males. Shape variation associated with CV1 and CV2 is shown by wireframe changes in relation to consensus configuration.
